# Sodium-glucose cotransporter 2 (SGLT2) inhibitor initiation and hepatocellular carcinoma prognosis

**DOI:** 10.1371/journal.pone.0274519

**Published:** 2022-09-12

**Authors:** Michael Hendryx, Yi Dong, Jonas M. Ndeke, Juhua Luo

**Affiliations:** 1 Department of Environmental and Occupational Health, School of Public Health, Indiana University, Bloomington, Indiana, United States of America; 2 Indiana University Simon Cancer Center, Indianapolis, Indiana, United States of America; 3 Division of Hematology Oncology, Department of Medicine, Indiana University School of Medicine, Indianapolis, Indiana, United States of America; 4 Department of Epidemiology and Biostatistics, School of Public Health, Indiana University, Bloomington, Indiana, United States of America; Texas A&M University, UNITED STATES

## Abstract

**Introduction:**

Sodium-glucose cotransporter 2 (SGLT2) inhibitors are a relatively new class of antidiabetic drugs. Emerging findings from laboratory studies indicate that SGLT2 inhibitors can improve liver function and suppress the proliferation of hepatocellular carcinoma (HCC) cells. The aim of this study was to test the hypothesis that initiation of SGLT2 inhibitors improves HCC prognosis in a human population.

**Methods:**

We used National Surveillance, Epidemiology and End Results (SEER)—Medicare linked data in the United States to evaluate the role of SGLT2 inhibitor initiation on the survival of HCC patients. 3,185 HCC patients newly diagnosed between 2014 and 2017 aged 66 years or older with pre-existing type 2 diabetes were included and followed to the end of 2019. Information on SGLT2 inhibitor initiation was extracted from the Medicare Part D file.

**Results:**

SGLT2 inhibitor initiation was associated with significantly lower mortality risk after adjusting for potential confounders (HR = 0.68, 95% CI = 0.54–0.86) with stronger association for longer duration of use (HR = 0.60, 95% CI = 0.41–0.88). Further, we found that SGLT2 inhibitor initiation was associated with a lower risk mortality risk ranging from 14% to 60% regardless of patient demographic variables, tumor characteristics, and cancer treatments.

**Conclusion:**

Our large SEER-Medicare linked data study indicates that SGLT2 inhibitor initiation was associated with improved overall survival of HCC patients with pre-existing type 2 diabetes compared with no SGLT2 inhibitor use. Further studies are needed to confirm our findings and elucidate the possible mechanisms behind the association.

## Introduction

Hepatocellular carcinoma (HCC) is the most common liver malignancy. Its incidence has increased in many countries in recent decades [1]. In the United States, incidence of HCC tripled from 1980 to 2015, although it appears to have stabilized in recent years [2]. The major risk factors for HCC vary from region to region. While the key determinants of HCC are chronic hepatitis B or C virus infection, aflatoxin exposure, and heavy alcohol consumption in most high risk HCC areas (such as China), excess body weight, type 2 diabetes (T2DM) and nonalcoholic fatty liver disease (NAFLD) are increasingly replacing viral- and alcohol-related causes as major pathogenic promotors in western countries, including the US [3–7].

Surgical resection, ablation or liver transplantation are curative treatments for eligible HCC patients with early-stage disease; however, most patients with HCC are diagnosed at an advanced stage. The 5-year survival rate for HCC is only 35% even for about 43% of patients diagnosed with localized stage disease, making HCC one of the deadliest cancers in the US [2]. Given the global rise in obesity, T2DM and other metabolic disorders represent the fastest growing cause of HCC in many parts of world, including the US [8]. Based on SEER-Medicare linked data, 59.6% of patients aged 68 years or older with HCC diagnosed between 1994 and 2007 had pre-existing T2DM [9]. T2DM is associated with poor prognosis of HCC [10]. However, little is known about how diabetes treatments affect HCC prognosis.

Sodium-glucose co-transporter (SGLT2) inhibitors are oral medications that are used to treat hyperglycemia in type 2 diabetes by blocking reabsorption of glucose in the renal proximal tubules, thereby promoting urinary glucose excretion [11, 12]. Four SGLT2 inhibitors are now commercially available in the US including canagliflozin, dapagliflozin, empagliflozin, and ertugliflozin [13]. Canagliflozin (brand name Invokana) was the first SGLT2 inhibitor approved by the Food and Drug Administration (FDA) in March 2013 [14]. Although less widely used than metformin, over 3 million canagliflozin prescriptions for diabetes treatment were written in the US in 2019 [15]. Besides their benefits in treatment of diabetes, SGLT2 inhibitors are considered to have additional renal and cardiovascular risk reduction benefits [16–18]. Because of these benefits, SGLT-2 inhibitors are recently being used one of the first-line drugs for glucose lowering, particularly in patients with type 2 diabetes at increased cardiovascular risk [19].

Accumulating animal studies and human clinical trial studies have reported that SGLT2 inhibitors may be effective for improvement of NAFLD [19, 20]. Several laboratory studies have also indicated that SGLT2-inhibitors have anti-proliferative activity by attenuating the uptake of glucose in several tumor cell lines, including HCC cells [21–25]. For example, Kaji et al. [25] conducted a xenograft study of human liver cells and observed that canagliflozin directly inhibited the growth of SGLT2-expressing liver cancers by reducing glucose uptake. Similarly, other laboratory studies have also suggested that SGLT2 inhibitors may have benefits for liver cancer [26–30].

These findings from laboratory and early clinical studies suggest that SGLT2 inhibitors may offer a potential new strategy for the treatment of HCC, especially for patients with T2DM. The beneficial impacts of SGLT2 inhibitors on HCC may include regulating metabolic reprograming, blocking glucose uptake by cancer cells, promoting weight loss, reducing inflammation or decreasing oxidative stress [25, 26, 31, 32]. However, to date, there are no population level epidemiological studies investigating this exciting possibility. If evidence is found in a large, heterogeneous, clinical population that SGLT2 inhibitors improve cancer survival, the impact is potentially profound, especially for liver cancer patients with limited treatment options and very poor prognosis. The current study uses US national Surveillance, Epidemiology, and End Results (SEER)-Medicare data to test the hypothesis that SGLT2 inhibitors may be associated with better HCC prognosis.

## Methods

### SEER-Medicare data

We used SEER—Medicare linked data for this study. The SEER program includes population–based tumor registries that routinely collect information on all newly diagnosed cancer cases that occur in persons residing in SEER areas, which cover approximately 48% of the US population.[33] The federally funded Medicare health insurance program covers people aged 65 years and older, people under age 65 with certain disabilities, and people of all ages with End-Stage Renal Disease (permanent kidney failure requiring dialysis or a kidney transplant).[34] Linking Medicare data with cancer registry data provides detailed information about Medicare beneficiaries with cancer and offers a unique population-based resource that can be used for epidemiological and health services research.[35] Based on a previous study, 95% of persons age 65 and older in the SEER files were matched to the Medicare enrollment file.[36] As of 2021, the data included all Medicare eligible persons who were diagnosed with cancer through 2017, and their Medicare claims through 2019.[35] The study was reviewed and approved by the Human Research Protection Program (HRPP) Office of Research Compliance–Indiana University, Protocol #: 2012984604. This is a secondary data analysis of deidentified data. Thus, no written informed consent was needed.

### Study population

**Fig 1:**
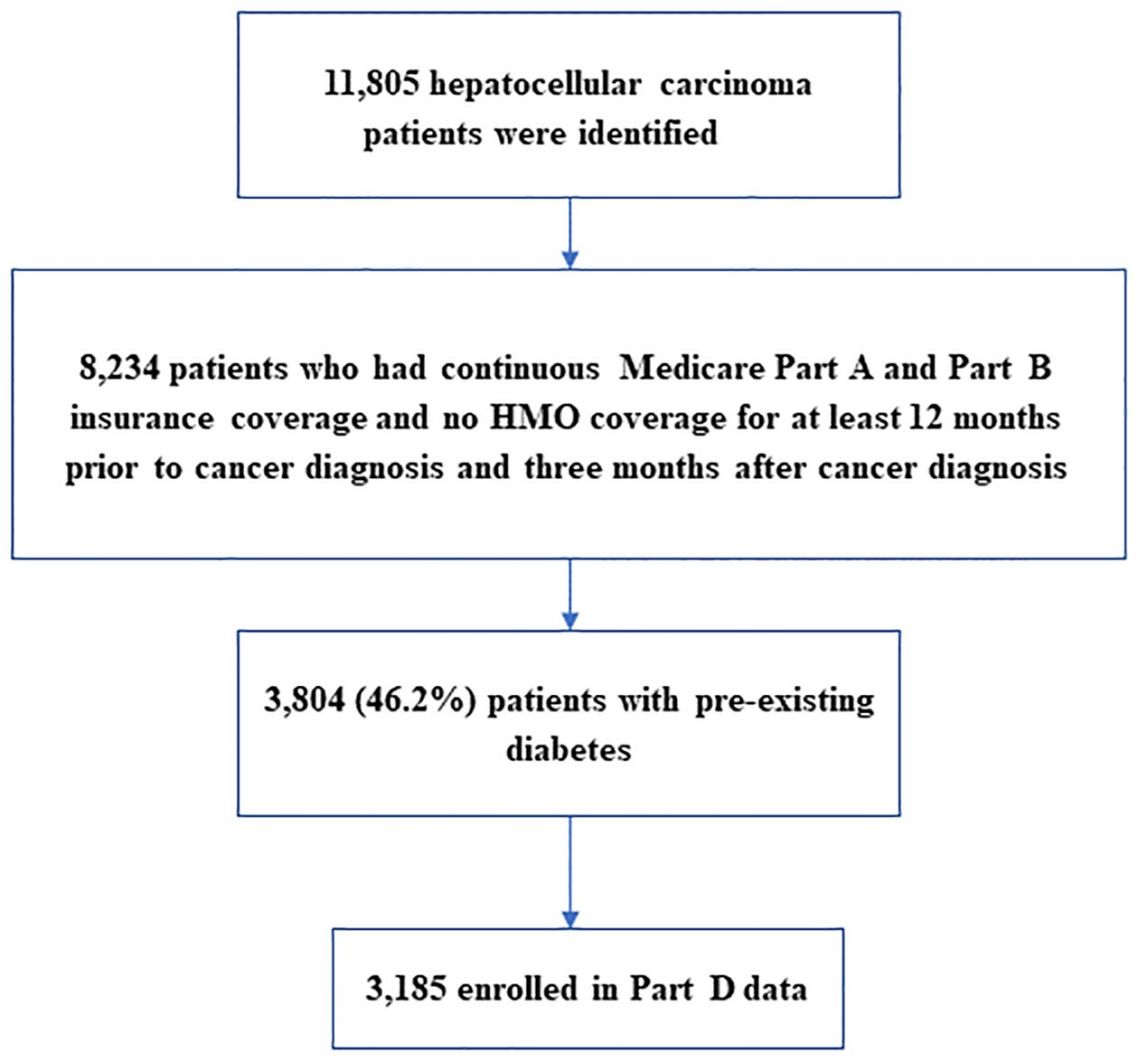
Study population flow chart.

We extracted all hepatocellular carcinoma (HCC) cases as a primary cancer diagnosed between 2014 and 2017 from the SEER-Medicare dataset. The cases were identified using the International Classification of Diseases (ICD-9 and ICD-10) for Oncology, Third Revision code C22 and morphology codes (8170–8175). Cases classified as benign or in-situ cancers were excluded from analysis. We restricted our study population to those with continuous Medicare Part A and Part B insurance coverage and no HMO coverage for at least 12 months prior to cancer diagnosis and 3 months after cancer diagnosis. The reason for this inclusion criterion is to entail 12 months coverage of prior cancer diagnosis for assessment of diabetes status and other comorbidities, and at least three months coverage of post cancer diagnosis for tracking of Medicare treatment choices and diabetes treatment. Among an initial sample of 11,805 HCC patients identified, 8,234 (69.8%) met inclusion criteria for continuous Medicare coverage. Of the 8,234 eligible patients, 3,804 (46.2%) patients with pre-existing diabetes were selected, and of these, 3,185 patients who were enrolled in the Part D event file (the prescription drug event data) remained for final analysis. The diabetes status in Medicare data was determined based on either a single inpatient claim or at least 2 outpatient claim ICD-9 or ICD-10 diagnoses separated by at least one day during the interval beginning 2 years before HCC diagnosis. A study population flow chart is shown in Fig 1.

### Exposures

Information on SGLT2 inhibitor initiation was extracted from the Part D event file, including canagliflozin, dapagliflozin, empagliflozin, and ertugliflozin. We also extracted information from the same Part D file on dipeptidyl peptidase 4 (DPP-4) inhibitors (another class of oral antidiabetic drugs) as an active comparator in a sensitivity analysis and information on metformin use as a confounder. The duration of SGLT2 inhibitors use was estimated in months as the sum of any SGLT2 inhibitor use during the follow-up period and by HCC diagnosis. For another sensitivity analysis (described below) we estimated the distance in months between initiation of SGLT2 inhibitors and cancer diagnosis for patients who initiated SGLT2 after cancer diagnosis, and re-set the distance to zero for patients who initiated SGLT2 inhibitors before cancer diagnosis because our follow-up started at cancer diagnosis.

### Outcomes

All-cause mortality was our primary outcome. The mortality data were extracted from Medicare claims data as of the end of 2019. The survival time was measured as months from date of cancer diagnosis to death or to the end of 2019 if the patient survived, whichever came first.

### Covariates

#### Tumor and treatment characteristics

HCC cancer stage was extracted from the SEER cancer file. Cancer stage was categorized as: localized (confined to primary site); regional (spread to regional lymph nodes); and distant (cancer has metastasized). Cancer treatment information was determined using both the SEER cancer file and Medicare data, including cancer-directed surgery, chemotherapy, and radiation. Although the SEER program routinely collects information regarding certain anti-cancer therapies (i.e., surgery, radiation therapy) occurring within 4 months of diagnosis, the sensitivity of using SEER data to identify these treatments is moderate [37]. Thus, we combined both datasets to identify cancer treatment and defined a patient to have a specific type of treatment if either database indicated yes to the type of treatments.

*Comorbidities*. Chronic conditions, including chronic kidney diseases, hypertension, and cardiovascular disease were based on the chronic condition flags file from the claims data. The Office of Enterprise Data and Analytics within the Center for Medicare and Medicaid Services (CMS) has developed pre-defined indicators for chronic conditions among Medicare beneficiaries enrolled in both Part A and Part B. A Medicare beneficiary is considered to have a chronic condition if the CMS administrative data have a claim indicating that the beneficiary received a service of treatment for the specific condition. Detailed information on the identification algorithms of the chronic conditions are available in the CMS Chronic Conditions Data Warehouse website [38]. Additional comorbidity covariates included diagnoses for cirrhosis, hepatitis B virus infection, hepatitis C virus infection, and alcohol-related diseases. These diagnoses were considered for the period of time from one year before cancer diagnosis to six months after diagnosis [39–41].

***Other covariates*** included patient’s demographic characteristics (age in years, sex, race or ethnicity (including non-Hispanic White, non-Hispanic Black or African American, non-White Hispanic, or Asian/Pacific Islander), marital status (married or not)) from SEER, metformin use, and diabetes duration (<5 years, 5-<10 years, 10 or more years).

### Statistical analysis

First, distribution of patients’ characteristics, tumor characteristics and treatment were compared between patients with and without SGLT2 inhibitor use. Chi-square tests were used to evaluate differences for categorical covariates, and t-tests were used for continuous variables.

Second, multivariable Cox proportional hazards regression models were used to estimate adjusted hazard ratios for mortality in relation to SGLT2 inhibitor initiation and duration of use adjusting for potential confounders including age, gender, race or ethnicity, comorbidities, metformin use, tumor stage and cancer treatment (age in continuous, others were categorized as listed in Table 1). For duration of SGLT2 inhibitor use, we only analyzed the duration before cancer diagnosis to minimize possible reverse causality.

**Table 1:** Patients’ characteristics by sodium-glucose cotransporter 2 (SGLT2) inhibitor use status among 3,185 hepatocellular carcinoma patients*.

	Use SGLT inhibitor use N (%)			
	Total (3185)	No (3048)	Yes (137)	P value
Age at diagnosis (years), mean (std)	74.8 (6.48)	74.9 (6.52)	72.5 (5.20)	<0.0001
Survival time, mean (std)	20.4 (17.6)	20.1 (17.5)	27.1 (17.4)	<0.0001
Diabetes duration (years), mean (std)	7.7 (4.81)	7.8 (4.80)	7.2 (5.00)	0.19
Sex				0.93
Male	2175 (68.29)	2081 (68.27)	94 (68.61)	
Female	1010 (31.71)	967 (31.73)	43 (31.39)	
Race/ethnicity				0.27
Non-Hispanic White	1624 (50.99)	1555 (51.02)	69 (50.36)	
Non-Hispanic Black	311 (9.76)	-	-	
Asian or Pacific Islander	518 (16.26)	488 (16.01)	59 (11.11)	
Hispanic/Latino	684 (21.48)	656 (21.52)	52 (9.79)	
Others	48 (1.51)		-	
Marital Status				0.18
No	1004 (32.72)	1004 (32.94)	38 (27.74)	
Yes	1205 (37.83)	1143 (37.50)	62 (45.26)	
Missing	938 (29.45)	901 (29.56)	37 (27.01)	
Chronic conditions				
Chronic kidney disease (yes, %)	1395 (43.80)	1323 (43.41)	72 (52.95)	0.03
Hypertension (yes, %)	2957 (92.84)	2833 (92.95)	124 (90.51)	0.28
Cardiovascular disease (yes, %)	2143 (67.28)	2053 (67.36)	90 (65.69)	0.69
Cancer stage				0.17
Localized	1773 (55.67)	1685 (55.28)	88 (64.23)	
Regional	824 (25.87)	797 (25.98)	32 (23.36)	
Distant	341 ((10.71)	-	-	
Missing	247 (7.76)	-	-	
Cancer treatment				
Surgery (yes, %)	776 (24.36)	726 (23.82)	50 (36.50)	0.0007
Chemotherapy (yes, %)	1274 (40.00)	1225 (40.19)	49 (35.77)	0.30
Radiation (yes, %)	807 (25.34)	767 (25.16)	40 (29.20)	0.29
Metformin use (yes, %)	1477 (46.37)	1374 (45.08)	103 (75.18)	<0.0001
Hepatitis C virus infection (yes%)	823 (25.84)	789 (25.89)	34 (24.82)	0.78
Hepatitis B virus infection (yes, %)	605 (19.00)	583(19.13)	22 (16.06)	0.37
Alcohol-related diseases (yes, %)	1142 (35.86)	1093 (35.86)	49 (35.77)	0.98
Liver cirrhosis (yes, %)	2183 (68.54)	2089 (68.54)	94 (68.61)	0.99

*—Numbers shown by—have been suppressed to protect confidentiality because of small cell counts.

Third, subgroup analyses were performed by patients’ demographic characteristics (age, sex, race or ethnicity), tumor characteristics, and tumor treatment. Finally, we performed four sensitivity analyses to assess the potential biases and robustness of our findings: (1) Applied propensity scoring among SGLT2 inhibitor user and non-users and then matched the two groups by propensity score with a caliper of 0.04 standard deviation of propensity score to further minimize confounding. (2) Applied an active comparator (DPP-4 inhibitor use) rather than non-SGLT2 inhibitor use. Similarly, we employed propensity scoring between SGLT2 inhibitor use and DPP-4 inhibitor use, and then matched the two groups by propensity score with a caliper of 0.12 standard deviation of propensity score. (3) For using DPP-4 inhibitors as an active comparator, in the matched step, we further matched the distance between the date of HCC diagnosis and initiation of two classes of drugs (±3 months). (4) Cause of death information was not provided in the Medicare claims data but was available for a shorter follow-up time through 2017 in SEER; cause of death data was available in 74% of cases (N = 2,357). We conducted a multivariable Cox proportional hazards regression model with death specifically from liver cancer as the outcome.

## Results

Of 3,185 HCC patients with pre-existing diabetes, 137 (4.3%) patients used SGLT2 inhibitors. Over an average of 20.4 months of follow-up, there were 2,426 (76.2%) patients who died in total. 78 patients died out of 137 who used SGLT2 inhibitors (crude mortality = 56.9%) while 2,348 died out of 3,048 patients who did not use SGLT2 inhibitors (crude mortality = 77.0%).

Table 1 presents a summary of patients’ characteristics according to SGLT2 inhibitor status. Compared to non-users, persons who used SGLT2 inhibitors were more likely to be younger, have chronic kidney disease and receive surgery, and have higher metformin use. No significant differences were observed for other characteristics.

Table 2 summarizes the associations between SGLT2 inhibitor use, duration, and mortality among patients with HCC. After adjusting for covariates, SGLT2 inhibitor use was significantly associated with 32% lower risk of mortality (HR = 0.68, 95% CI = 0.54–0.86). Stratified analysis by duration of use showed that compared with no use, longer duration (12 months or more) of SGLT2 inhibitor use was significantly associated with more pronounced reduced mortality (HR = 0.60, 95% CI = 0.41–0.88) (Table 2).

**Table 2:** Associations between sodium-glucose cotransporter 2 (SGLT2) inhibitor use and mortality among 3,185 hepatocellular carcinoma patients with T2DM.

	Number of deaths/Number of patients	HR (95% CI) *
**SGLT2 inhibitor use**		
No	2348/3048	Reference
Yes	78/137	0.68 (0.54–0.86)
Duration of use		
<12 months	51/85	0.73 (0.55–0.97)
≥ 12 months	27/52	0.60 (0.41–0.88)

*Models were adjusted for age at diagnosis, sex, race/ethnicity, marital status, chronic conditions (chronic kidney disease, hypertension, cardiovascular disease), cancer stage, cancer treatment (cancer-directed surgery, chemotherapy, radiation), diabetes duration (<5 years, 5-<10 years, 10 or more years), metformin use, hepatitis C virus infection, hepatitis B virus infection, alcohol-related diseases, and cirrhosis.

**Fig 2:**
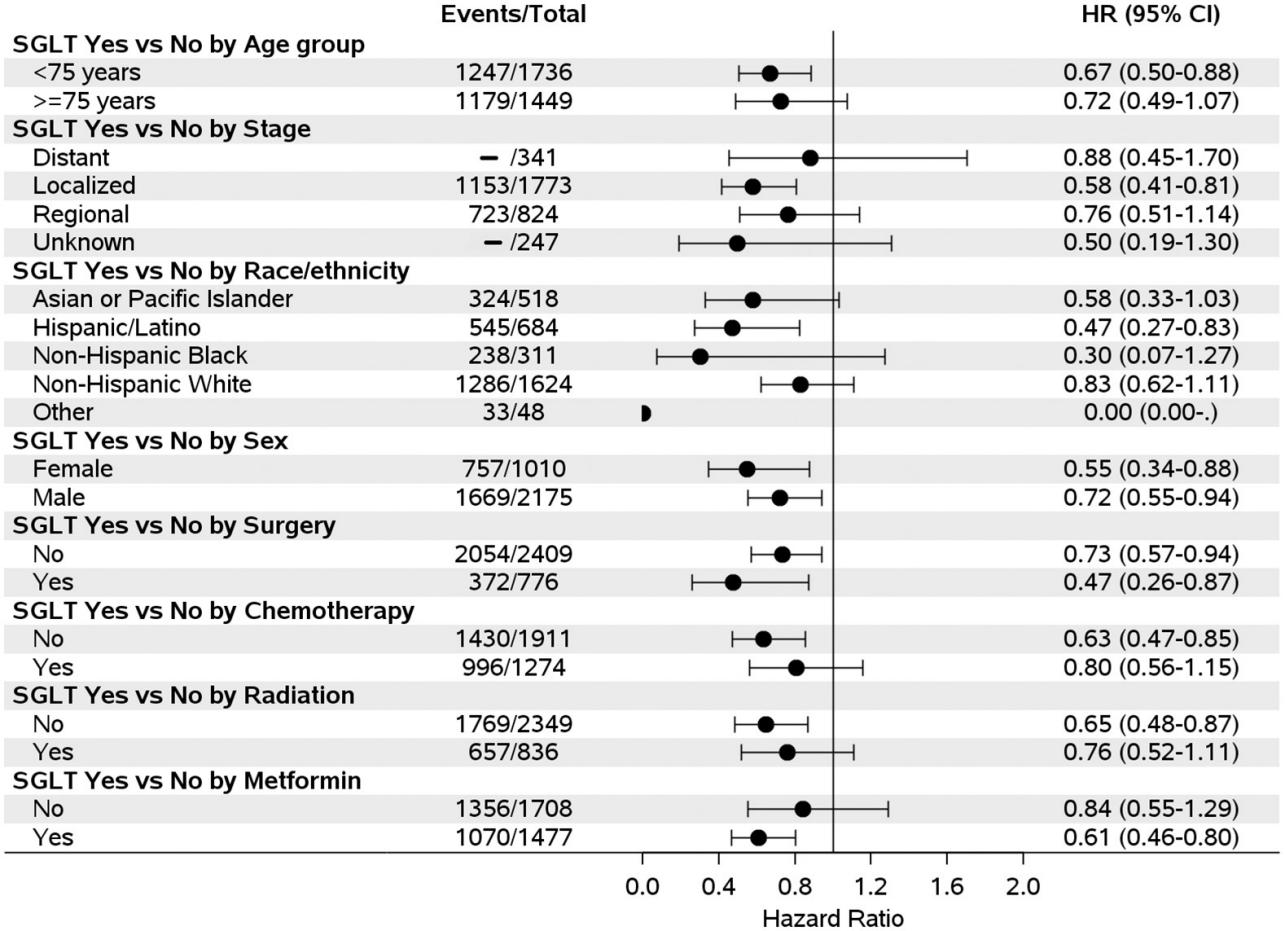
Forest plot of association between sodium-glucose cotransporter 2 (SGLT2) inhibitor use and hepatocellular carcinoma mortality by covariates (- Numbers have been suppressed to protect confidentiality).

We further examined the association between SGLT2 inhibitor use and risk of mortality stratified by different covariates and found that SGLT2 inhibitor use was associated with a reduced risk of mortality regardless of patients’ demographic, tumor characteristics and cancer treatments, although some results did not reach statistical significance due to small sample sizes in some of the sub-groups (Fig 2).

Sensitivity analyses showed that reduced risk of mortality remained associated with SGLT2 inhibitor use. In sensitivity analysis 3 that employed the propensity score approach and further matched distance between HCC diagnosis and initiation of the two compared drugs, SGLT2 inhibitor use was not statistically significant and long duration of SGLT2 inhibitor use was only borderline significant (Table 3). The final sensitivity analysis specific to liver cancer as cause of death from SEER confirmed the findings: SGLT2 inhibitor use was associated with reduced risk of mortality (HR = 0.58, 95% CI = 0.42–0.89) and the association was stronger for longer duration of use (HR = 0.37, 95% CI = 0.19, 0.71.).

**Table 3:** Associations between sodium-glucose cotransporter 2 (SGLT2) inhibitor use and mortality among hepatocellular carcinoma patients with T2DM*.

	Sensitivity analysis 1		Sensitivity analysis 2		Sensitivity analysis 3	
	Deaths/Patients	HR (95% CI)	Deaths/Patients	HR (95% CI)	Deaths/Patients	HR (95% CI)
**SGLT2 inhibitor use**						
No	86/137	Reference	88/137	Reference	77/122	Reference
Yes	78/137	0.50 (0.37–0.69)	78/137	0.78 (0.57–1.08)	74/122	0.78 (0.55–1.10)
Duration of use						
<12 months	51/85	0.52 (0.37–0.75)	51/85	0.89 (0.62–1.29)	48/67	0.86 (0.58–1.29)
≥ 12 months	27/52	0.47 (0.30–0.74)	27/52	0.63 (0.40–0.998)	26/55	0.66 (0.41–1.07)

*Models were adjusted for age at diagnosis, sex, race/ethnicity, marital status, chronic conditions (chronic kidney disease, hypertension, cardiovascular disease), cancer stage, cancer treatment (cancer-directed surgery, chemotherapy, radiation), diabetes duration (<5 years, 5-<10 years, 10 or more years), metformin use, hepatitis C virus infection, hepatitis B virus infection, alcohol-related diseases, and cirrhosis.

Sensitivity analysis 1 –employed propensity score among SGLT2i users and non-users and then matched the two groups by propensity score with a caliper of 0.04 standard deviation of propensity score.

Sensitivity analysis 2 –employed propensity score among SGLT2i users and DPP4i users and then matched the two groups by propensity score with a caliper of 0.12 standard deviation of propensity score.

Sensitivity analysis 3 –employed propensity score among SGLT2i users and DPP4i users and then matched the two groups by propensity score with a caliper of 0.12 standard deviation of propensity score and distance between HCC diagnosis and initiation of the two compared drugs (± 3 months).

## Discussion

Our study based on the SEER-Medicare linked data, a large population-based national database, revealed that HCC patients with pre-existing T2DM treated with SGLT2 inhibitors had better prognosis relative to patients who were not on SGLT2 inhibitors, especially for those who used SGLT2 inhibitors ≥ 12 months. We further observed that SGLT2 inhibitor use was associated with a reduced risk of mortality regardless of patients’ demographic, tumor characteristics and cancer treatments, despite some associations in sub-groups not reaching statistical significance. The associations remained significant when limiting cause of death specifically to liver cancer.

Our study is the first to examine the role of SGLT2 inhibitors on HCC prognosis in humans based on a large population-based national database. The potential benefits of SGLT2 inhibitors for liver cancer have been reported in several *in-vitro* and *in-vivo* laboratory studies [25–30]. For example, four studies [25–27, 29] and reported that canagliflozin suppressed the proliferation of HCC cells. Kaji et al. [25] also observed canagliflozin directly reduced human HCC xenograft tumor growth. Furthermore, two *in-vivo* studies [27, 28] used mouse models of diabetes and NASH related hepatocarcinogenesis and observed fewer hepatic tumors in the continuous canagliflozin use group compared with control group. Obara et al. [30] also observed that the development of hepatic preneoplastic lesions was markedly suppressed in mice treated with tofogliflozin, although liver cancer cell growth was not directly suppressed.

The mechanisms by which SGLT2 inhibitors may contribute to better prognosis of HCC are unclear. Several possible explanations have been proposed. First, SGLT2 inhibitors may have a direct inhibitory effect on the occurrence and progression of HCC [27]. Studies have reported that SGLT2 inhibitors suppressed HCC proliferation by inducing G2/M arrest and/or promoting apoptosis of HCC cells [25–27]. Studies have also reported that SGLT2 inhibitors can exert antiproliferative effects by regulating metabolic reprograming and blocking glucose uptake by cancer cells [25, 26]. For example, Kaji observed that canagliflozin exerted antiproliferative effects on SGLT2-expressing Huh7 and HepG2 cells in a dose-dependent manner by inhibiting glycolytic metabolism [25]. Second, SGLT2 inhibitors may contribute to better prognosis of HCC via improved liver function and preventing the development of hepatocarcinogenesis from NAFLD. SGLT2 inhibitors have been reported to ameliorate hepatic steatosis in patients with diabetes and non-alcohol fatty liver disease [19, 42]. Recent studies have suggested that the stage of fibrosis is the most important predictor of liver outcomes, including HCC [43–45]. Studies have shown that SGLT-2 inhibitors are able to attenuate liver fibrosis, improve liver biochemical parameters, reduce fatty liver content (a surrogate of hepatic steatosis), and decrease liver histopathology by attenuating hepatic lipid accumulation and inducing fatty acid oxidation enzyme and autophagy [46, 47]. These reported benefits on liver function may be due to glucose-lowering and weight loss effects promoted by SGLT2 inhibitors, and may be independent of glycemic control and/or weight loss [48, 49], such as though improvement of insulin resistance, reduction of inflammatory markers and decrease in oxidative stress [31, 32]. Recent data have demonstrated that SGLT2 inhibitors can attenuate chronic inflammation and hepatic steatosis and protect tissues against oxidative damage not only by their glucose-lowering effects but also via either lowering free-radical generation or by boosting the biological antioxidative system [49–54].

Our study includes a number of strengths such as the analysis of a prospective, population-based, nationally representative database that contains rich co-morbidity, tumor characteristics, and cancer treatment data important for studying prognosis. However, several limitations should be recognized. First, analysis of medication effects may be complicated by changes in therapy. Diabetes progression or evolving approaches to optimal management might influence diabetes treatment regimens over time, making it difficult to disentangle the effects of variable lengths of exposure to different drug combinations. We only analyzed initiation of SGLT2 use and accumulated length of SGLT2 inhibitor use by cancer diagnoses and did not assess different drug combinations. Second, confounding by indication is a potential concern because patients receiving SGLT2 inhibitors may have different clinical characteristics than patients receiving other diabetes treatments such as metformin. However, SGLT2 inhibitors may be more likely to be prescribed for advanced diabetes after metformin failure, which might render our findings conservative. Further, we performed several sensitivity analyses by employing a propensity score approach and using an active comparator. Although most results in the sensitivity analyses were similar, results were attenuated after further matching the distance between HCC diagnosis and initiation of the two compared drugs, indicating that potential survival bias could not be ruled out. Third, we did not have data on health behaviors such as body mass index (BMI) or physical activity. Thus, we were not able to account for these potential confounders in the analysis. Fourth, we were not able to distinguish between curative and non-curative radiation treatments, which might have important implications for survival outcomes, although we note that radiation treatment overall did not differ between groups. Fifth, Medicare claims data did not include cause of death, so we were limited to a subset of data with the shorter follow-up time available from SEER for analysis of cancer-specific mortality.

In conclusion, our findings based on a large SEER-Medicare database indicate that HCC patients with pre-existing T2DM treated with SGLT2 inhibitors had significantly lower risk of mortality, especially among those treated more than 12 months. Associations were robust when SGLT2 inhibitor use was tested relative to an active comparator. Further studies are needed to confirm our findings and elucidate the possible mechanisms behind the association.
